# Epilepsy*Read at meeting of Louisville Medico-Chirurgical Society, February, 1894.

**Published:** 1894-04

**Authors:** J. A. Larrabee

**Affiliations:** Professor of Diseases of Children in the Hospital College of Medicine, Louisville, Ky.


### Southern Medical Record.

A MONTHLY JOURNAL OF MEDICINE AND SURGERY.

VOL. XXIV. ATLANTA, GA., APRIL, 1894. No. 4.

### Original Articles.

### EPILEPSY.*

BY J. A. LARRABEE, M.D.,
Professor of Diseases of Children in the Hospital College of Medicine,
LOUISVILLE, KY.

Notwithstanding the fact that epilepsy as a disease has been described by the oldest medical writers, and that it is in all probability coeval with the oldest history of man, we are as unable to-day to demonstrate its pathology and indicate its cure as when two thousand three hundred and eighty years ago it was graphically described by the great "father of medicine." The most comprehensive answer to the question, What is epilepsy? is that it is an explosion of nerve electrical force, or, in a word, vaso-motor spasm.

With the exception of those instances where traumatism has established an irritating cause, the brain of the epileptic dissected after death shows no lesion which could be made to account for the seizures. While there is a marked leaning of medical authorities to heredity as a predisposing cause, there is very great reason to doubt, and statistics by no means confirm such a theory.

In regard to chronic alcoholism on the part of either parent, statistics are more conclusive and leave very little room to doubt that

*Read at meeting of Louisville Medico-Chirurgical Society, February, 1894.

drunkenness stands in the relation of cause and effect in the production of epileptic children.

Syphilis, that protean Hydra of the human race, has also made claims which are not proven. It cannot be doubted that acquired syphilis is productive of serious organic lesions of the nerve system, but epilepsy is not one of these.

That parents of highly nervous organization, hysterical mothers and neurotics in general, are likely to transmit to their offspring a diathesis favorable to the development of epilepsy is entirely agreeable to what we know of diabetes and other neuroses.

Statistics gathered from asylums and hospitals show that one-third of all cases of epilepsy in children have begun before the tenth year of life, and that three-fourths of all before the twentieth year.

Hippocrates nearly three thousand years ago wrote these words concerning epilepsy: "The cure may be attempted in young persons, but not in old." This paper is based upon the analysis of twenty cases of well established epilepsy, covering a period of seventeen years, and ranging in age from five to twenty-five. All have been cured, or in other words have been entirely free from *grand* or *petit mal* since treatment, but the recent cases cover now only two and one year observation. Of course these still remain doubtful. One case, a girl of ten years, who had as many as five or six *grand mal* seizures a month, had a severe spell last September after a whole year of perfect immunity. She is not included in the above number. It is interesting to note that this attack was undoubtedly brought about by a hearty meal of sausage and pork. The mother remembered, better than I, that I had said in my lectures that the child could be allowed to eat meat at the end of the year if she had no spells. On the very anniversary of that day she gave the above menu for supper. She has again been put on treatment, and has had no return up to now.

The first case, seventeen years ago, was an adult who was obliged to give up a lucrative position by reason of the frequency of his attacks. He had been treated for years unsuccessfully by bromides, and presented the bromidean acne when he came to me. He remained two years under treatment, and is now in California, and at last accounts was well. He was treated with sulphate of copper, and had the copper line well marked upon the gums. Six other adults have been discharged and remain well. One treated by gold has had no spells for six months. The remainder were children at and below the age of puberty. One patient still under treatment with chloride of gold and sodium, although markedly benefited, has been obliged to suspend the treatment on account of bowel and stomach derangement. From my limited experience I have found the salts of zinc and gold to be more irritating than ammonia sulphate of copper, which I prefer.

I believe that nitrate of silver in small doses continued long enough to produce argyria will result in the cure of ninety per cent. of epilectics. But the disfiguration is of such a character that it would be unwarrantable practice without a written contract in which the patient agrees to look all through his life like a highly polished stove-pipe. Believing as I do that the metals tin, copper, zinc and gold all act in the same manner, namely, storage in connection with the nerve cells in the discharging areas of the brain, I have used them with the above results.

Two of my epileptic patients, not included in the above list, have died in a fit; both could have been saved had any one been present. One, a most estimable lady, was found face down upon the bed; the other fell into a tanner's vat where he was employed. No epileptic should be allowed to sleep on a feather pillow.

In speaking of my treatment of these cases I claim nothing new or original. The therapeutics of epilepsy has included at different periods in the history of medicine nearly every drug and chemical in the *materia medica*, nor is it strange that a disease, whose pathology is shrouded in mystery, should, in ancient times, have been the subject of all that is mythical, mysterious, and superstitious in medicine. Not only has the earth and sea been scoured for remedies, and vile and disgusting concoctions made from the animal and vegetable kingdom, but the planetary system has been invoked and the subtle salts of the alchemists have had their day, and, what is stranger still, their cures. The charm and the amulet, the mistletoe gathered under the exacting rules of the Druids, have all cured under the dominant idea.

*Bromides.*—The introduction of bromic acid united with various bases marks an important era in infantile therapeutics. This is the "Sweet oblivious antidote" dreamed of by the immortal "Bard of Avon" with which we may "erase the writ tablets of the brain." While the bromides are inadequate to the cure of confirmed epilepsy, they are of all agents the best, to obtund the brain to peripheral impressions. Their sphere of action is in the prevention rather than in the cure, and some one of the salts of bromine should be continued for two weeks after a fit of eclampsia in the infant or child. A longer continuation is apt to produce unpleasant symptoms of bromism. Ammonia is the best base for any of the acids, therefore preferable to potash. In adults these agents, carried to their toxical effect, secure often long immunities to a seizure, but only so long as a condition bordering upon imbecility lasts. With a cessation of the drug and a return of the nerve centers to their pristine vigor, the attacks return in full force. It is quite different with the child, and some permanent cures may be expected in the growing brain from the administration of bromides.

*Nocturnal Epilepsy, Night Terrors or Nightmare.*—The fact that epilepsy may exist for a long time unobserved is important. When a strong healthy man or woman is heard to utter a cry and seen to fall to the ground in a convulsion, attention is attracted to them. When a child stops for a moment at its play and fixes its eyes upon some object, remaining immovable for a moment of time, and then without any other manifestation resumes its play as though nothing had occurred, no attention is paid to it. Such attacks, however, constitute the "*petit mal*" of epilepsy. "Not infrequently children awaken in the morning complaining of violent headache and the soiled and disarranged bedding afford the only evidence of a struggle concerning which the child has no recollection." Such instances are by no means common, and their true nature is only revealed by some fellow occupant who is put to watch them.

The relation of night terrors of children to epilepsy is also a matter of very great importance. This distressing condition, often "poohed" at and less frequently but too often made the subject of chastisement, has a marked effect upon the growing brain and forms the background in the history of many an adult epileptic. Children who by inheritance are possessed of a neurotic diathesis are more prone to such attacks.

When such authorities as Hennock, of Berlin, and Goodheart, of London, make the statement that they do not consider food to be a factor of any prominence in the etiology of night terrors, it may seem presumptuous for me to compare my limited experience and limited opportunities of experience with their extensive observation. Nevertheless an honest difference of opinion, however humble the source, is offered in the spirit of truthful investigation. From that experience I am forced to conclude that not only certain foods, but also the time at which they are taken, has largely to do with the production of night terrors and consequently with the development of epilepsy in childhood. I have met with very few where the night terrors were not traceable to ingesta in the intestinal tract, and with none in which a regulation of diet, especially the last meal, did not inaugurate an improvement and an amelioration of the night attacks.

Nitrogen is an essential element in gunpowder and other explosives with the destructive force of which we are only too familiar. It is scarcely less dangerous and destructive when circulating to an excess in the blood of animals whose nerve force it has the power of suddenly discharging. Epilepsy may be produced at will in the young of even carnivorous animals by feeding raw meat during the period (formative) of lactation. Kittens so fed will develop fits in a few days which prove fatal if continued, but which cease with the withdrawal of the meat. Horses overfed with stimulating, nitrogenous food, have epilepsy and fall trembling in the harness. A piece of raw beefsteak with tea or coffee at supper will be as equally certain to cause an uproar in the nursery during the night.

It is a suggestive fact that the brightest minds that have adorned our nation, and the greatest statesmen who have figured in history, were put to bed as children upon bread and milk or oaten porridge. Whether or not the "Meat and Morals" of the great Russian reformer "Tolstoi" shall receive scientific indorsement is yet to be seen, but quiet sleep and brain rest in children are both incompatible with meat and tea and coffee. Nine-tenths of all the various convulsive manifestations in infancy and childhood have their origin in intestinal irritation or from a loaded bowel, packed cæcum or intestinal worms.

I can remember when I used to prescribe bromide, chloral, sulphonal etc., for the muscular, gastrocnemius cramps of elderly people with some temporary benefit. Latterly, however, I have found much in common between childhood and age. I find that an acid condition of the jejunum and ileum from faulty assimilation of faulty food is the cause of the acidity, and I have also found that a simple alkali, as soda bicarb., taken on retiring, secures exemption from the trouble.

The intellectual status of confirmed epileptics is often a matter of astonishment when we take into consideration the apparent gravity of the seizures. When an explosion occurs in one of the discharging areas of the brain the senses are under shock, and a condition like concussion occurs in the centers of conscious thought. Nearly always before the suspension of consciousness, the victim ejaculates a word or sentence, which word or sentence remains peculiar to the individual, and then immediately the memory is obliterated. Often the aura is through one of the special senses, as vision, smell, or peripheral irritation. The same object is seen, the same odor is smelled and the same peripheral zone is felt before each seizure. My experience among children leads me to indicate more frequently an ocular spectacle which is remembered as a ray of light approaching and blinding the victim. As regards the vigor of the intellect of epileptics, it must be admitted that in the growing age it is impaired by the attacks and often results in imbecility, while in age memory becomes almost entirely obliterated and the various manifestations of softening occur.

The fact that some of the world's greatest masters have been life-long epileptics must not be forgotten. The greatest epochs in history have been inaugurated by epileptics, while poets have sung and martyrs have suffered under its strange hallucinations. Upwards of a hundred millions of the world's population to-day bow the knee as devotees to the erratic religious devotions of an epileptic, whose prophetic visions and vagaries were but a part of epilepsy. The old Roman wall of England, the broken arches upon the Rhine, the lofty peaks of Cisalpine Gaul bear silent witness to the indomitable will, fortitude and genius of an epileptic who lived before the Christian era; while in the still remembered years of modern history we recall the burning of Moscow, the carnage of Austerlitz and Jena, and the culminating battle of Waterloo as the work of an epileptic whose gigantic intellect was extinguished amid the tempest of that awful night at St. Helena.

Persons who develop epilepsy after the age of puberty appear to retain their mental vigor in the interval of the attacks, and are often capable of attending to all the duties of life. They are moreover possessed of strong will power and determination. If a religious trend has been given to their character, they become very devout, often fanatical, and make sacrifices which would be quite impossible for ordinary individuals.

Confirmed epileptics often enjoy immunity to attacks by a preoccupancy of the mind or by engagements in hazardous undertakings, *e. g.*: A famous church spire painter who worked in Louisville was an epileptic, and could be seen any day suspended upon his airy platform one hundred and fifty feet above the ground. He never had a spell while aloft pursuing his work, but once back upon *terra firma* he would be seized with a fit. So marked is this feature that treatment by a new physician, especially if he be a celebrity, often secures a long immunity to the seizures. The knowledge of this fact should caution us against attributing the relief to our therapeutic measures.

Epileptics seldom die in a paroxysm but often live long and useful lives. A gentleman well known in this city and throughout the State, whose Christian example and good works will be handed down to future generations, has been an epileptic from boyhood. He was refused life insurance over forty years ago and has outlived every one of the agents and physicians of the company. He has been twice paralyzed, first right and then left hemiplegia, and is to-day a sound reasoner and thinker.

I desire to call especial attention to the importance of the treatment of infantile eclampsia. The commonly accepted fact that convulsions—epileptiform—are of frequent occurrence in inverse proportion to age, may possibly be the cause of carelessness in general practice, the consequences and the importance of a complete restoration of the nerve centers is forgotten. The foundation for future epilepsy is thus laid. This is about the way it runs: The family physician is called to a child in a fit; he arrives—is informed that it is all over—the child has had a spasm but is now sleeping quietly, if he wishes he can come in and see it. Now, instead of explaining to the friends the importance of after-treatment, care, diet and possible medication, he is too apt to say: "Oh, never mind; I guess it will be all right, and if he has any more trouble let me know."

The increasing tendency to convulsions in infancy may be attributed to two principal causes, namely: first, the more perfect development of the animal brain—cerebellum; second, the immature development of the intellectual brain—cerebrum.

All the acts and functions of the new-born babe are reflex, rhythmical, only becoming manifest with the deposit of gray matter, *e. g.*: Cheyne-Stokes respiration denotes serious disease in a child and adult, but it is the normal respiration of an infant. The pulse has no regularity, and noise and slight concussion are responded to, as in a frog poisoned by strychnine. Gradually, however, the will power, "that column of true majesty in man," develops and asserts its power over the spinal system. Just in proportion to this development, or non-development, an infant remains liable to convulsions. Every practicing physician knows of families possessed of such a diathesis, and learns that a hasty summons on account of some trifling ailment means a spasm. A child who has had a convulsion from any of these causes is more liable to have another from a lesser cause, and so on, until finally a "status epileptica" is established, after which the fits occur without any apparent cause. We know comparatively little of the process by which such photographic impressions are made upon the growing brain of the infant, but we do know that the stains are well-nigh indelible.

